# Estrogen Withdrawal-Induced Cognitive Impairment in Menopausal Women: Mechanisms and Prospects for Integrated Interventions

**DOI:** 10.3390/ijms27157003

**Published:** 2026-08-04

**Authors:** Tiantian Qiu, Junying Zhang, Jiayou Zhao

**Affiliations:** Institute of Basic Research in Clinical Medicine, China Academy of Chinese Medical Sciences, Beijing 100700, China; 17812090810@163.com

**Keywords:** estrogen withdrawal, cognitive dysfunction, mitochondrial, neuroimmunity, NLRP3 inflammasome, estrogen receptor beta

## Abstract

A marked reduction in estrogen levels during perimenopause substantially elevates the risk of Alzheimer’s disease (AD) and cognitive dysfunction in women. While the endocrine etiology is well established, applying this understanding to effective clinical prevention remains difficult. Recent findings of diminished cerebral glucose metabolism and lower mitochondrial cytochrome oxidase activity in menopausal women have shifted research attention toward mitochondrial homeostasis disruption and neuroimmune–inflammatory network imbalance as central mechanisms underlying menopausal cognitive decline. This article examines the characteristics and underlying mechanisms of mitochondrial and immune imbalances induced by estrogen withdrawal during menopause. Estrogen deficiency is shown to disrupt mitochondrial–immune homeostasis, particularly via ERβ-mediated mitochondrial oxidative phosphorylation system (OXPHOS) dysfunction and subsequent excessive activation of the NLRP3 inflammasome. The analysis further addresses enhanced inflammatory signaling resulting from excessive reactive oxygen species generation and mitochondrial DNA (mtDNA) release, as well as reduced synaptic plasticity due to impaired neurotransmitter synthesis and an inflammatory microenvironment. Additionally, the dysregulation of the estrogen-neuromodulatory system in menopausal cognitive decline is investigated. Recent studies demonstrate that intervention strategies targeting estrogen receptors, especially selective ERβ agonists, possess significant neuroprotective potential. Future approaches should incorporate biomarkers, including neuroimaging and genetic polymorphisms, to facilitate risk-stratified and individualized precision medicine. This integration may enhance the prevention or delay of menopause-associated cognitive decline in women.

## 1. Introduction

Natural menopause typically occurs in women between 45 and 51 years of age [1]. It is characterized by the absence of menstruation for 12 consecutive months, without identifiable pathological or physiological causes. The perimenopausal period is the interval before menopause, typically lasting 5 to 8 years, during which women transition from reproductive maturity to menopause. It is characterized by a gradual decline in ovarian function and fertility [2]. Perimenopause involves significant endocrine remodeling, with characteristic hormonal changes such as the gradual decrease and fluctuation of ovarian hormones, particularly estradiol and progesterone. These hormonal imbalances are linked to an elevated risk of osteoporosis, diabetes, stroke, cardiovascular disease, sleep disorders, cancer, cognitive decline, and Alzheimer’s disease (AD) [3,4,5,6].

Approximately one in five women develop AD by the age of seventy, and sex hormones are thought to contribute to gender differences in its pathogenesis and progression [7,8,9]. The pronounced decline in estrogen levels during perimenopause may represent a critical period for therapeutic intervention [10]. Recent multimodal brain imaging studies have compared cognitively normal perimenopausal and postmenopausal women aged 40 to 60 years with age- and education-matched men. These studies demonstrate that menopause is associated with the emergence of multiple imaging markers characteristic of the AD endophenotype, including reduced frontal cortex glucose metabolism, increased β-amyloid accumulation, and decreased gray and white matter volume [11]. Reduced brain metabolism correlates with decreased platelet mitochondrial cytochrome oxidase activity, indicating that perimenopausal and postmenopausal women experience bioenergetic deficits comparable to those observed in AD [12]. Additional research indicates that systemic inflammation and declining estrogen levels during perimenopause may contribute to Aβ accumulation. Moreover, the Apolipoprotein E4 (APOE4) genotype, through its interaction with estrogen, elevates the risk of AD in women relative to men [13,14,15]. Understanding the biological mechanisms underlying the perimenopausal transition may facilitate a reduction in AD risk in women and inform the development of strategies to mitigate menopause-related health complications.

Estrogen is a steroid hormone synthesized from cholesterol and exhibits diverse biological activities. Its primary forms are estrone (E1), estradiol (17β-estradiol, E2), and estriol (E3). E2 is the main circulating estrogen during the reproductive period and has the highest physiological activity. E2 deficiency disrupts the synthesis and signaling of key neurotransmitters, including acetylcholine, glutamate, and neuroprotective peptides. This disruption results in reduced cerebral perfusion and vasoconstriction, which decreases oxygen delivery to brain tissue and exacerbates cognitive impairment. Numerous studies have demonstrated that E2 mediates neuroprotective and regulatory effects in the brain by activating estrogen receptor subtypes α (ERα), β (ERβ), and the G protein-coupled estrogen receptor 1 (GPER-1; also known as GPR30) [16]. ERα and ERβ are broadly distributed within emotion-related neural circuits, exhibiting especially prominent activity in the ventral cortical–limbic–brainstem axis [17,18]. ERα predominantly regulates nuclear gene transcription and reproductive neuroendocrine processes. In contrast, ERβ is primarily localized to the mitochondrial membrane, where it modulates mitochondrial metabolism and cellular stress responses, thereby exerting a critical influence on diverse neurobiological functions [19,20]. Research indicates that the concentrations of ERα and ERβ in the brain vary throughout the aging process [21]. As age increases, both ERα and ERβ levels decline in the synapses of the hippocampal CA1 region in rats. However, in contrast to ERα, ERβ expression increases in aged rats following estrogen stimulation [21,22]. This review examines the mechanisms through which estrogen deficiency contributes to cognitive decline in women undergoing the menopausal transition.

## 2. The Biological Basis of Endocrine Transition

**Figure 1:**
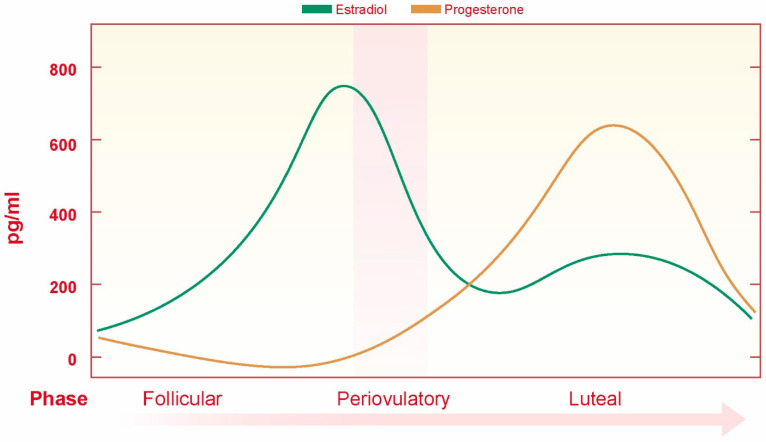
Changes in estradiol and progesterone concentrations throughout the female menstrual cycle (follicular phase, periovulatory phase, and luteal phase). The green solid line represents estradiol (E2), and the orange solid line represents progesterone. The horizontal red arrow at the bottom indicates the sequential progression of three menstrual cycle stages from left to right (follicular → periovulatory → luteal). The vertical axis shows hormone concentration with the unit pg/mL, while the horizontal axis labels the three distinct menstrual phases.

The secretion of estrogen and progesterone in females is regulated by the hypothalamic–pituitary–gonadal axis. The gonadotropin-releasing hormone, produced by the hypothalamus, stimulates the anterior pituitary to release luteinizing hormone and follicle-stimulating hormones. Luteinizing and follicle-stimulating hormones collaboratively regulate the ovarian cycle. During the follicular phase, follicle-stimulating hormones stimulate follicle maturation and the release of estrogen. In the luteal phase, luteinizing hormones induce ovulation and stimulate the corpus luteum, which develops from the ruptured follicle, to secrete progesterone. Estrogen exerts feedback effects on the pituitary and hypothalamus to regulate the release of follicle-stimulating hormones, luteinizing hormones, and gonadotropin-releasing hormones. The interplay of feedforward and feedback mechanisms precisely coordinates the activity of the hypothalamic–pituitary–gonadal axis, thereby establishing a normal menstrual cycle [23]. E2 levels fluctuate throughout the female menstrual cycle during the reproductive period (Figure 1). Following menopause, serum estradiol levels decrease markedly from 5–35 ng/dL to approximately 1.3 ng/dL. This pronounced reduction is recognized as a significant biological event that contributes to emotional instability and cognitive dysfunction during the menopausal transition, thereby altering the functioning of emotion regulation and cognitive systems influenced by these hormones.

## 3. Effects of E2 Deficiency and Supplementation on Cognitive Function

E2 exerts both genomic and non-genomic effects. The non-genomic effects mediated by mitogen-activated protein kinase (MAPK) activation may contribute to adult synaptic plasticity [24,25]. Studies employing gene-knockout animals have demonstrated that ERα, ERβ, and GPR30/GPER1 mediate hippocampal synaptic plasticity as well as the cognitive effects of estrogen [26]. Sequential activation of ERα and ERβ induces synaptic plasticity through interactions with metabotropic glutamate receptors [27]. Selective activation of ERα increases dendritic spine density and enhances cognitive ability, while selective activation of ERβ does not alter dendritic spine density and only improves cognition in specific tasks at certain doses [28]. Selective activation of GPR30/GPER1 induces synaptic plasticity in the hippocampus [29]. These findings indicate that ERα, ERβ, and GPR30/GPER1 each have the potential to regulate cognitive processes. In addition to ERα, ERβ, and GPR30/GPER1, studies have identified two further membrane-localized estrogen receptors in the central nervous system, which are associated with G protein coupling [30]. Regulation of E2 levels is critical for mediating synaptic morphological changes across multiple preclinical species. In rodents, ovariectomy results in a reduction in dendritic spines, particularly on apical CA1 pyramidal cells, while E2 treatment reverses this effect. Research in rhesus monkeys has shown that natural menopause leads to a selective loss of perforated synapses in the outer molecular layer of the dentate gyrus, which are essential for sustaining long-term synaptic potentiation. Subsequent research by the same group demonstrated that E2 treatment increases synaptic density in the prefrontal cortex of aged ovariectomized rhesus monkeys [31]. Additionally, following ovariectomy in aged rhesus monkeys, a reduction in multisynaptic boutons in the dorsolateral prefrontal cortex was observed, which E2 treatment can reverse [32]. Magnetic resonance imaging (MRI) studies in clinical populations have demonstrated that E2 treatment increases hippocampal volume in postmenopausal women in a dose-dependent manner. Cognitive and imaging endpoint assessments further indicate that greater cognitive decline in postmenopausal women correlates with increased connectivity in the executive control network and temporal lobes, as well as decreased connectivity in the frontal lobe [33]. Functional MRI assessments using multiple paradigms indicate that postmenopausal women treated with E2 exhibit improved brain functional efficiency during sustained attention tasks. Additionally, in working memory tasks, frontal lobe activation increases with task difficulty following E2 treatment [34]. The maintenance of synaptic plasticity and neurotransmitter synthesis is highly dependent on cellular energy supply. Recent studies demonstrate that the subcellular localization of estrogen receptors is closely associated with mitochondria, which play a central role in cellular energy metabolism. These findings provide a more comprehensive cell biological perspective for understanding cognitive impairment induced by estrogen deficiency.

## 4. Mitochondrial Dysfunction as a Central Link Between Estrogen Deficiency and Cognitive Impairment

Mitochondria are primary sites for the biosynthesis of steroid hormones, including estrogen. Studies have demonstrated that specific estrogen receptors (ERs) are localized within the mitochondria of the frontal cortex and hippocampus. Recent mechanistic proposals identify mitochondrial dysfunction as a central pathological factor. Mitochondria are essential organelles responsible for ATP production and are critical in the regulation of synaptic plasticity, calcium homeostasis, oxidative stress and apoptosis [35]. Disruption of mitochondrial homeostasis, defined by impaired bioenergetic synthesis, elevated reactive oxygen species (ROS) production, and heightened inflammatory signaling, compromises neuronal structure and function, particularly in brain regions critical for emotional and cognitive functions such as the hippocampus and prefrontal cortex [36,37]. An expanding body of evidence indicates that mitochondrial dysfunction is linked to emotional and cognitive impairments in perimenopausal women.

Studies indicate that the marked reduction in E2 levels during menopause disrupts mitochondrial homeostasis and produces measurable bioenergetic changes in both central and peripheral tissues. Clinical studies indicate that ATP production efficiency in postmenopausal women decreases by approximately 30 percent, a reduction directly linked to diminished activity of mitochondrial complex IV, cytochrome c oxidase. Translational neuroimaging studies utilizing ^18^F-fluorodeoxyglucose positron emission tomography (PET) and ^31^P magnetic resonance spectroscopy (MRS) indicate that the metabolic rate of glucose consumption in the brains of perimenopausal women declines concurrently with reduced efficiency of oxidative phosphorylation [38]. Moreover, reduced cerebral glucose utilization correlates with decreased cytochrome c oxidase activity in peripheral blood platelets, indicating concurrent mitochondrial metabolic dysfunction in both the central nervous system and peripheral tissues. This metabolic disruption is attributed to both chronological and endocrine aging, the latter being a hormone-mediated process that destabilizes mitochondrial function. These findings suggest that mitochondrial dependence on E2 represents a key mechanistic factor contributing to cognitive decline during perimenopause.

Beyond the bioenergetic phenotype, this mitochondrial dysfunction triggered by estrogen deficiency exerts profound downstream impacts on cognitive function by impairing neuronal viability, disrupting synaptic plasticity, and promoting the accumulation of AD pathological proteins [39]. Estrogen deficiency has been shown to induce mitochondrial damage that precedes the emergence of cognitive deficits, directly linking E2 withdrawal to neuronal bioenergetic failure and subsequent cognitive decline [39]. Neurons are highly polarized cells with immense ATP demands to maintain membrane potential and action potential propagation; accordingly, perturbations in mitochondrial ATP production compromise neuronal excitability and synaptic transmission [40]. The mitochondrial energy failure induced by E2 deficiency compromises Na^+^/K^+^-ATPase and Ca^2+^ pumps, leading to ion imbalances and excitotoxicity [41,42]. Furthermore, the loss of mitochondrial Ca^2+^ buffering capacity exacerbates neuronal stress, ultimately triggering apoptotic pathways and neuronal loss, especially in vulnerable regions like the hippocampus [42]. Synaptic plasticity, encompassing synaptogenesis, maintenance, and remodeling, comprises energy-demanding processes in which mitochondria supply the local ATP required for presynaptic vesicle priming and neurotransmitter metabolism [43]. Mitochondrial dysfunction reduces presynaptic ATP availability for vesicle cycling and neurotransmitter release, including acetylcholine and glutamate [44]. Postsynaptically, energy deprivation impairs receptor trafficking and dendritic spine maintenance [45]. Studies indicate that ovariectomy-induced loss of circulating 17β-estradiol disrupts mitochondrial respiration and promotes oxidative stress-mediated degradation of synaptic proteins within the entorhinal cortex–hippocampal circuitry. These synaptic mitochondrial deficits correlate with impaired long-term potentiation (LTP) and reduced dendritic spine density in the hippocampal CA1 region, effects reversible by E2 treatment via restoration of mitochondrial distribution and energy supply [46]. Furthermore, mitochondrial dysfunction plays a pivotal role in initiating and accelerating AD-related pathological proteins. Impaired mitochondrial complexes (e.g., cytochrome c oxidase) increase ROS production, which promotes the aberrant cleavage of the amyloid precursor protein (APP) and favors β-amyloid (Aβ) accumulation [47]. In turn, Aβ exerts toxic effects on mitochondria at synaptic sites, creating a vicious cycle [48]. Additionally, oxidative stress and energy failure activate glycogen synthase kinase-3β (GSK-3β), promoting Tau hyperphosphorylation and contributing to neurofibrillary tangle formation [49]. Thus, the mitochondrial decline induced by E2 withdrawal is not merely a bioenergetic phenotype but a critical trigger for the neuropathological cascades driving cognitive decline.

Elucidating how estrogen orchestrates these mitochondrial processes to forestall such decline has centered on specific receptor-mediated pathways. Previous studies have demonstrated that ERβ is localized within neuronal mitochondria and contributes to the regulation of mitochondrial function [50]. Mitochondrial estrogen receptors play a direct role in the estrogen-mediated maintenance and regulation of mitochondrial structure and function [51,52]. The mitochondrial oxidative phosphorylation system (OXPHOS) resides in the inner mitochondrial membrane and comprises five multimeric complexes (complexes I–V or CI–CV). The biosynthesis of the OXPHOS depends on both mitochondrial and nuclear genomes [53]. Estrogen receptors bind to estrogen response elements within the D-loop region of mitochondrial DNA, indicating a role in regulating mitochondrial gene expression [54]. Studies using a human mammary epithelial cell line have shown that the E2-induced increase in mRNA levels of mitochondrial DNA-encoded cytochrome c oxidase subunits I and II is inhibited by the estrogen antagonist fulvestrant (ICI), indicating that this process depends on the estrogen receptor [55]. In an ovariectomized rat model, mRNA levels of cytokine concentration subunit III in the hippocampus increased significantly within three hours of E2 administration [56]. The localization of estrogen receptors in both nuclear and mitochondrial compartments suggests that these receptors regulate mitochondrial biosynthesis and function through nuclear–mitochondrial signaling interactions. Further in vitro evidence supports this signaling interaction by demonstrating the shuttling of ERβ between mitochondria and the nucleus [57]. ERβ possesses a mitochondrial localization sequence, whereas ERα lacks this feature. Unlike mechanisms that directly regulate mitochondrial DNA, studies have shown that mitochondrial ERβ influences cellular processes through CREB phosphorylation. This process enables phosphorylated CREB to bind directly to the D-loop region of mitochondrial DNA and regulate the expression of OXPHOS subunit genes [58]. Evidence indicates that, following E2 treatment, silencing ERβ leads to reduced levels of phosphorylated CREB in both the nucleus and mitochondria. These findings suggest that ERβ plays a critical role in CREB phosphorylation within these subcellular compartments [50]. Moreover, ERβ silencing reduced the protein levels of mitochondrial-encoded complex IV subunits 1, 2, and 3, thereby confirming the involvement of ERβ in phosphorylated CREB-mediated mitochondrial OXPHOS protein expression [50].

The nuclear receptor ERRα, which shares homologous DNA sequences with ERα, also plays a significant role in regulating mitochondrial function. ERRα regulates transcription by interacting with the estrogen signaling pathway or by binding to co-responsive elements on target genes. Numerous studies have shown that ERRα regulates mitochondrial function, mitochondrial turnover, and lipolysis [59,60]. Protein expression levels of ERRα are significantly reduced in the cerebral cortex of 6-month-old APP/PS1 mice, indicating dysregulation of ERRα in AD [61]. Furthermore, ERRα knockout mice exhibit atypical feeding and social behaviors. Ning’s findings indicate that in SH-SY5Y cells treated with Aβ oligomers, overexpression and activation of ERRα are sufficient to rescue mitochondrial dysfunction. ERRα agonists also restore cognitive deficits in AD mice induced by intracerebroventricular injection of Aβ1–42 [62]. Collectively, these results suggest that APOE2-ERRα-dependent regulation of mitochondrial function is a mechanism involved in the pathological process of AD [62]. APOE subtypes interact with ERRα. APOE is proposed to stimulate ERRα translocation into the cell nucleus, where ERRα functions as a transcription factor to activate downstream genes and regulate mitochondrial function. The overexpression of APOE2 leads to increased ERRα expression, indicating that ERRα could represent a potential target for the prevention and treatment of AD.

In summary, estrogen deficiency fundamentally disrupts the structural and functional integrity of neuronal mitochondria, the functional state of which is tightly regulated by ERβ and related receptors such as ERRα. This bioenergetic crisis extends beyond mere phenotypic alterations to directly impair neuronal survival, cripple the energy-dependent mechanisms indispensable for synaptic plasticity, and facilitate the accumulation of AD-like pathologies, including Aβ aggregation and Tau hyperphosphorylation. Consequently, mitochondrial dysfunction serves as the crucial pathological bridge linking the endocrine shifts in the menopausal transition to long-term cognitive decline and neurodegeneration in women. 

## 5. Mitochondrial and Microenvironmental Contributions to the Neuroimmune Inflammatory Network Induced by Estrogen Deficiency

Estrogen serves as a central regulator of immune and inflammatory processes. In comparison to men, postmenopausal women demonstrate heightened inflammatory responses to infections, an increased incidence of autoimmune diseases, and fluctuating activity levels of chronic inflammatory diseases associated with the menopause, menstrual cycle, and pregnancy. Estrogen has been demonstrated to positively regulate the maintenance of naive B cells, which in turn promotes the development of humoral immune responses. Furthermore, estrogen reduces peripheral blood levels of the pro-inflammatory cytokine IL-6, inhibits the secretion of inflammatory cytokines by CD4+ T cells, and alleviates inflammatory responses in perimenopausal women [63]. The presented evidence suggests that estrogen corrects immune imbalances during the perimenopausal period and supports the maintenance of immune homeostasis. An analysis of peripheral blood lymphocyte subsets in postmenopausal and reproductive-age women indicates that postmenopausal women exhibit a reduced total lymphocyte count. Notably, both B lymphocytes and CD4+ T cells are significantly decreased, and the CD4+/CD8+ T cell ratio is also substantially lower. This reduction ultimately results in an inverted CD4+/CD8+ T cell ratio, which is a hallmark of aging associated with increased oxidative stress [64,65,66]. Kumru’s study demonstrated that E2 reduces the number of CD8+ T cells, restores the peripheral blood CD4+/CD8+ T cell ratio to normal levels, and significantly increases the proportion of CD19+ B cells and interferon-γ levels [67]. E2 restores the proportions of various lymphocyte subsets in perimenopausal women. However, the counts of CD4+ T cells and CD20+ B cells remain lower than those in women of reproductive age. These findings indicate that estrogen is essential for regulating the immune status of perimenopausal women; however, it does not counteract the age-related decline in immune function [63]. Immune system alterations in postmenopausal women increase susceptibility to inflammation. Evidence indicates that women who undergo oophorectomy prior to menopause have higher levels of the inflammatory marker C-reactive protein than those who have a hysterectomy with ovarian preservation. Malutan conducted a comparative analysis of inflammatory cytokine levels among women of reproductive age, perimenopausal women, postmenopausal women, women following oophorectomy, and women with chronic inflammation. The study found that postmenopausal women exhibited significantly higher levels of the inflammatory cytokines IL-1β, IL-8, IL-6, IL-4, and tumor necrosis factor-α, whereas levels of the anti-inflammatory cytokine IL-20 were lower [68]. Elevated inflammatory markers tend to return to baseline levels after sex hormone therapy. Studies using rat models of inflammation have demonstrated that ERβ downregulates the P2X3 receptor in peripheral tissues, resulting in an anti-inflammatory effect [69]. In peripheral inflammation models involving female rodents, cytokine concentrations in the brains of ovariectomized subjects increased, while E2 treatment restored these concentrations to normal levels [70,71]. Postmenopausal increases in inflammatory cytokines and blood–brain barrier permeability indicate that a heightened systemic inflammatory state may contribute to cognitive abnormalities, with E2 serving as a critical anti-inflammatory factor. Chronic inflammation, characterized by glial cell preactivation, pro-inflammatory cytokine release, and neuronal damage, may also play a significant role in the progression of AD, a condition with increased risk among women [72].

Although the mechanisms underlying immune dysregulation in perimenopausal women remain incompletely understood [66], ongoing research utilizing perimenopausal animal models and relevant cell lines is progressively clarifying these processes. Estrogen receptors are specifically expressed in the cell membrane, cytoplasm, and nucleus of various cell types, including macrophages, monocytes, neutrophils, dendritic cells, natural killer cells, CD8+ and CD4+ T cells, B cells, regulatory T cells, astrocytes, microglia, and neurons [73]. Upon activation by estrogen, various estrogen receptors regulate gene expression either directly or in cooperation with transcription factors, and also modulate cellular signal transduction through second messengers such as cAMP. Recent investigations into immune imbalance associated with estrogen deficiency during perimenopause have focused on the regulation of inflammatory signaling pathways mediated by ERα, ERβ, and GPER [74]. Recent studies demonstrate that mitochondria are essential in regulating the innate inflammatory response mediated by the inflammasome protein NLRP3, which functions as a sensor for disturbances in homeostasis, including mitochondrial dysfunction [75]. A widely supported mechanism of NLRP3 activation involves the generation of mitochondrial ROS and the subsequent translocation of NLRP3 to the mitochondria, which results in the release of mitochondrial DNA [76]. NLRP3 activators induce mitochondrial destabilization, promote NLRP3 deubiquitination, facilitate linear ubiquitination of the inflammasome protein ASC, and trigger the exocytosis or release of mitochondrial-derived molecules, including mitochondrial DNA. These molecules interact with NLRP3 following its translocation to the mitochondria, resulting in activation of the NLRP3 inflammasome [77]. Recent studies indicate that mitochondria function as anchoring sites for NLRP3, thereby regulating inflammasome complex activity. Additionally, mitochondrial ROS intensify the immunogenic signaling of the inflammasome. In contrast, activation of mitochondrial autophagy reduces the inflammatory response by removing the NLRP3 complex associated with mitochondria [78]. Studies in NLRP3-knockout animals subjected to focal cerebral ischemia demonstrate significant reductions in both infarct volume and neurovascular damage, supporting the critical role of NLRP3 in ischemic pathophysiology [79]. Several studies have demonstrated that E2 suppresses the gene expression of the NLRP3 inflammasome in the cerebral cortex after focal cerebral ischemia [80,81]. The modulatory effects of estrogen on hippocampal inflammation, as well as depression- and anxiety-like behaviors, are mediated by ERβ [82,83]. However, the precise function of ERβ in regulating NLRP3 inflammasome activation within the central nervous system remains unclear. Activation of ERβ confers ischemic protection, stimulates mitochondrial function, and inhibits inflammasome activation, indicating a central role in the signaling interaction between the inflammasome and mitochondria. A more comprehensive understanding of these mechanisms may facilitate the development of novel therapeutic strategies.

**Figure 2:**
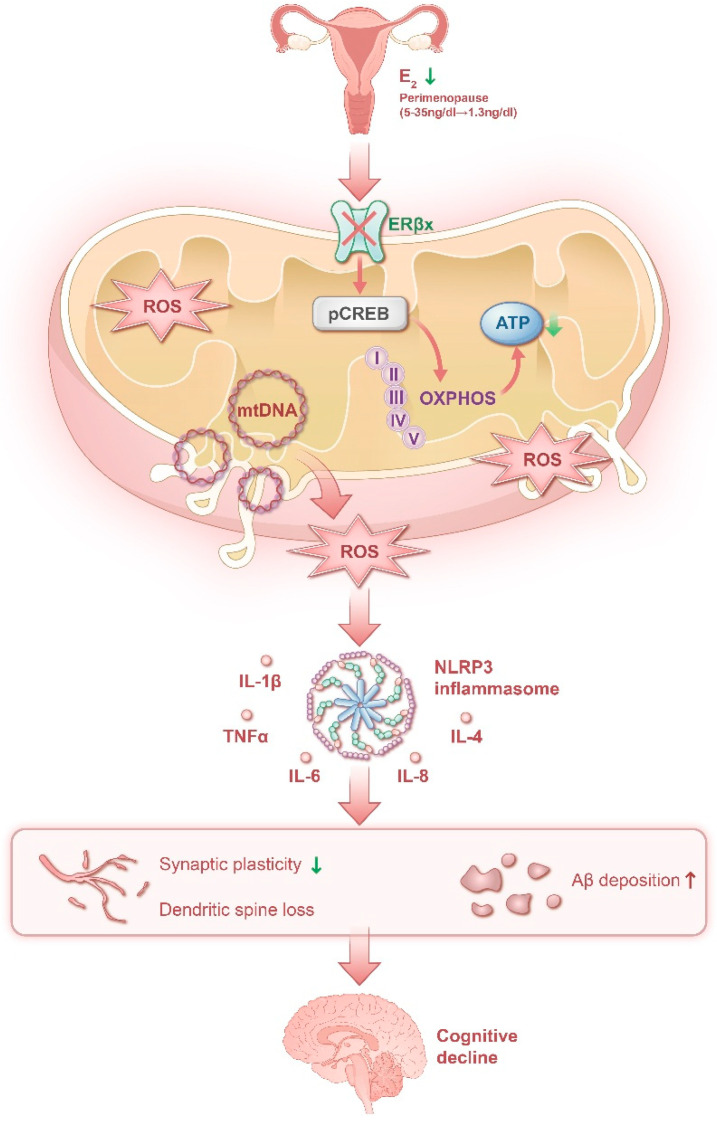
The mechanism by which estrogen withdrawal during the menopausal transition leads to cognitive dysfunction through the collapse of mitochondrial homeostasis and neuroimmune–inflammatory network imbalance. The marked decline in E2 during menopause disrupts ERβ-mediated mitochondrial oxidative phosphorylation (OXPHOS), leading to a bioenergetic crisis and increased reactive oxygen species (ROS) generation. Destabilized mitochondria subsequently release mitochondrial DNA (mtDNA) into the cytoplasm, and this, together with ROS accumulation, induces excessive activation of the NLRP3 inflammasome. This activation results in the release of pro-inflammatory cytokines, creating a microenvironment that intensifies neuroinflammation and promotes glial cell activation. The interplay of impaired bioenergetics, oxidative stress, and neuroinflammation disrupts neurotransmitter synthesis and diminishes synaptic plasticity, ultimately leading to cognitive decline associated with menopause. The green downward arrows indicate inhibitory effects or decreased levels, and red upward arrows represent elevated levels. The red cross (×) marks impaired ERβ signaling. Roman numerals (I–V) correspond to the five multimeric complexes of the mitochondrial oxidative phosphorylation system (OXPHOS) located on the inner mitochondrial membrane (complexes I–V).

In summary, the decline in estrogen levels during the menopausal transition drives a systemic inflammatory state. This state is characterized by increased systemic pro-inflammatory cytokines derived from reproductive tissues, alterations in the cellular immune profile, elevated inflammasome proteins within the central nervous system, and the formation of a pro-inflammatory microenvironment. Pro-inflammatory processes may weaken the protective effect of ERβ against ischemic brain injury and impair mitochondrial function, which regulates inflammasome activation. This sequence of events establishes a basis for the development of neurodegenerative diseases, including cognitive dysfunction, in women later in life. Collectively, these findings indicate the presence of a complex regulatory system that spans estrogen withdrawal, mitochondrial energy crisis, and neuroimmune inflammation, referred to as the estrogen-neuromodulatory system (Figure 2). Consequently, pharmacological agents targeting molecules within estrogen receptor signaling pathways may contribute to the intervention and treatment of menopausal cognitive dysfunction.

## 6. Therapeutic Strategies Targeting Estrogen Receptors

In preclinical models of menopause, ovariectomy in young rodents results in deficits in spatial learning tasks, such as the radial arm maze and the Morris water maze [84,85]. E2 supplementation reverses cognitive deficits in young and middle-aged ovariectomized rodents. In contrast, the effects of E2 supplementation in aged ovariectomized rodents are inconsistent; some studies report improvements in episodic memory, while others find no enhancement in working memory. Studies utilizing primary neuronal cultures have demonstrated that E2 reduces Aβ production and secretion. Furthermore, estrogen administration in estrogen-deprived mice reverses elevated Aβ levels in the brain [86]. Observational studies indicate that hormone therapy administered after menopause may reduce the risk of AD [87]. Additionally, a recent study demonstrated that transdermal E2 therapy reduced β-amyloid deposition in recently postmenopausal women [88], suggesting that E2 may contribute to the regulation of β-amyloid plaque formation. Clinical studies have shown that women treated with both acetylcholinesterase inhibitors and E2 exhibit greater improvements on cognitive assessments than those not receiving E2 therapy [89]. Gibbs demonstrated that E2 treatment alone did not ameliorate deficits observed in aged rats following ovariectomy. However, when E2 was administered in combination with donepezil, an acetylcholinesterase inhibitor, the capacity of E2 to enhance learning in the delayed matching task in aged rats was restored [90]. Similar results have been observed in aged rats treated with galantamine, another acetylcholinesterase inhibitor. The same study also demonstrated that E2 treatment enhances cholinergic release in the hippocampus [85]. Therefore, combining hormonal and neurotransmitter-based therapies may enhance the efficacy of both treatment strategies.

Research differentiating the effects of various receptors has demonstrated that ERα [27,91], ERβ, and GPR30/GPER1 each contribute to the regulation of cognitive function. G-1, a selective agonist of GPR30/GPER1, enhances the cognitive performance of ovariectomized animals in the delayed place-matching, novel object recognition, and Y-maze tasks [92,93]. Acetylcholinesterase inhibitors approved by the FDA represent the primary pharmacological treatment for AD. These agents increase acetylcholine concentrations in the synaptic cleft, thereby enhancing cholinergic signaling. Cellular studies have demonstrated that the G protein-coupled estrogen receptor GPR30/GPER1 colocalizes with neurons expressing choline acetyltransferase (ChAT), particularly within the basal forebrain [94]. Additionally, ERα colocalizes with ChAT-expressing neurons in the basal forebrain [95]. Previous research and clinical practice indicate that ERβ-selective agonists could serve as a safer and more effective therapeutic target in future studies compared to ERα agonists or E2 alone. Activation of ERβ in the brain confers ischemic protection, enhances mitochondrial function, and suppresses inflammasome activation. ERβ agonists are considered safer, as ERβ does not stimulate proliferation in mammary or endometrial tissues. Furthermore, ERβ agonists may exert concurrent effects on both the cardiovascular and cerebrovascular systems, thereby reducing ischemic injury. ERβ signaling represents a promising direction for future translational research focused on mitigating cognitive decline and cerebral ischemic events in postmenopausal women, while minimizing the adverse effects linked to prolonged E2 therapy.

## 7. Discussion

The perimenopausal period represents a critical transitional phase for women, marking the shift from reproductive maturity to menopause. This stage is characterized by fundamental endocrine remodeling, particularly the withdrawal of estrogen, which contributes to brain aging through multiple mechanisms. Understanding these processes offers important insights into sex differences in neurodegenerative diseases, including AD in women. Within the field of neuro-reproductive medicine, this article systematically reviews the primary mechanisms underlying cognitive impairment resulting from estrogen deficiency. Specifically, it examines the synergistic interaction between mitochondrial homeostasis disruption and the neuroimmune–inflammatory network. Additionally, the article discusses therapeutic strategies based on estrogen receptors, providing a theoretical foundation for future interventions targeting menopause-associated cognitive decline in women.

While preclinical data strongly support the neuroprotective potential of estrogen, translating these findings into effective clinical prevention of AD remains challenging. Inconsistent results across studies highlight the limitations of a uniform approach to hormone therapy [88]. Prevention strategies should be tailored to individual patients, considering both the timing of menopause and distinct risk phenotypes. Implementation of these strategies necessitates the integration of diverse biomarkers into clinical decision-making. Relevant biomarkers include neuroimaging indicators such as hippocampal atrophy, cerebral perfusion, and PET-detected tau and amyloid-β pathology [96,97]; plasma or cerebrospinal fluid biomarkers such as the Aβ42/40 ratio, phosphorylated tau, and neurofilament light chain; and genetic markers including apolipoprotein E (APOE) genotype and other AD-related risk polymorphisms [98,99]. In addition, combination therapeutic approaches involving estrogen demonstrate significant potential. For example, hormone therapy combined with antihypertensive or antidiabetic agents may reveal cognitive benefits by addressing vascular risk factors, while hormone therapy in conjunction with lifestyle interventions such as exercise, a Mediterranean diet, and cognitive training may further enhance brain plasticity [14]. Most positive effects on cognitive endpoints have been primarily observed during the menopausal transition period, which is also marked by significant brain structural remodeling and alterations in estrogen receptor expression. These findings indicate that clinicians should recognize menopause-associated cognitive impairment as an independent clinical entity. In the future, individualized or precision medicine, guided by factors such as age, timing, genotype, and vascular health, will likely become increasingly important. Compared to a universal hormone prescription strategy for all postmenopausal women, a refined risk stratification approach that identifies individuals at higher risk for late-life cognitive or mood disorders and selectively administers hormone therapy is expected to provide greater clinical benefits.

## Data Availability

No new data were created or analyzed in this study. Data sharing is not applicable to this article.

## Abbreviations

OXPHOS	oxidative phosphorylation system
mtDNA	mitochondrial DNA
AD	Alzheimer’s disease
E1	estrone
E2	estradiol
E3	estriol
ERs	estrogen receptors
ERα	estrogen receptor subtypes α
ERβ	estrogen receptor subtypes β
GPER-1/GPR30	G protein-coupled estrogen receptor 1
MAPK	mitogen-activated protein kinase
MRI	magnetic resonance imaging
ROS	reactive oxygen species
PET	positron emission tomography
MRS	magnetic resonance spectroscopy
ChAT	choline acetyltransferase
APOE	apolipoprotein E
HPG	hypothalamic–pituitary–gonadal
GnRH	gonadotropin-releasing hormone
LH	luteinizing hormone
FSH	follicle-stimulating hormone
